# A Prospective Observational Study to Determine Rate of Thromboprophylaxis in Oncology Patients Undergoing Abdominal or Pelvic Surgery

**DOI:** 10.1007/s13193-021-01281-0

**Published:** 2021-03-01

**Authors:** Shailesh V. Shrikhande, Manish Verma

**Affiliations:** 1 Department of Surgical Oncology, Tata Memorial Hospital, Ernest Borges Marg, Parel, Mumbai, 400012 India; 2 Medical Affairs, Sanofi, Mumbai, India

**Keywords:** Venous thrombosis, Observational study, Low-molecular-weight heparin, Ultrasonography, Doppler, Pelvic neoplasms

## Abstract

Venous thromboembolism (VTE) is common in patients with cancer and is a leading cause of death. In addition to the hypercoagulable state associated with malignancy, cancer-related surgery and subsequent immobilization further increase the risk of VTE. Guidelines suggest extended prophylaxis up to 4 weeks with low-molecular-weight heparin (LMWH) in such patients. This study is conducted to determine the proportion of patients receiving thromboprophylaxis among those undergoing surgery for malignant abdominal or pelvic tumor. This prospective, multicenter, observational study included 300 patients (217 [72.3%] were women). Mean age and duration of cancer were 53.2 and 1.2 years, respectively. A total of 162 (54%) patients received thromboprophylaxis of which only pharmacological in 78 [48.1%], only mechanical in 27 [16.7%], and both pharmacological and mechanical in 57 [35.2%] patients. LMWH (128, 79.0%) and graduated compression stockings (74, 45.7%) were the commonly used modalities. VTE prophylaxis was given in only half of the patients. Physician education to increase adherence to international guidelines is very important. Trial Registration No. CTRI/2013/05/003617.

**Supplementary Information:**

The online version contains supplementary material available at 10.1007/s13193-021-01281-0.

## Introduction

Venous thromboembolism (VTE) occurs in 4–20% cancer patients and is a leading cause of death in patients with cancer [1]. The risk of first VTE is approximately sevenfold higher in patients with malignancy than in those without it [2]. In Indian patients diagnosed with VTE, malignancy mainly abdominal and pelvic cancer is the most common predisposing factor [3]. Further, patients with cancer undergoing surgical procedures have twice the risk of postoperative VTE and more than three times the risk of fatal pulmonary embolism (PE) than patients who undergo surgery for benign diseases [4]. Recent studies show that incidences of symptomatic deep vein thrombosis (DVT) and PE among Asian colorectal cancer surgery patients are comparable to those in the West [5]. Likewise the prevalence of PE in cancer patients in India is not as low as originally assumed, and PE is associated with high mortality in these patients [6].

A significant percentage of morbidity and mortality in cancer patients can be attributed to hypercoagulation [7]. In addition to the hypercoagulable state associated with malignancy, cancer-related surgery, chemotherapy, and immobilization further increase the risk of VTE [7]. Despite the risk of VTE complications in patients with a malignancy, a large number of hospitalized patients undergoing major abdominal or pelvic surgery for cancer are still not prescribed appropriate thromboprophylaxis [8].

The American Society of Clinical Oncology (ASCO) guidelines recommend that patients undergoing major surgery for malignant disease should be considered for pharmacologic thromboprophylaxis, while patients undergoing major abdominal or pelvic surgery for cancer who have high-risk features, such as restricted mobility, should be administered extended prophylaxis with low-molecular-weight heparin (LMWH) for up to 4 weeks postoperatively [9, 10]. Similarly, the European Society of Medical Oncology (ESMO) guidelines (2011) state that cancer patients undergoing elective major abdominal or pelvic surgery should receive in hospital and post-discharge prophylaxis with subcutaneous LMWH for up to 1 month after surgery [11].

However, there are limited data available on whether these recommendations have impacted the real-world management of cancer surgery patients and led to changes in the routine clinical practice [12]. Although many trials have evaluated the efficacy and safety of anticoagulants for prophylaxis after major abdominal surgery, most of these trials included only a small proportion of patients who were undergoing surgery for cancer [13]. Further there are no India-specific data regarding the proportion of patients who are given thromboprophylaxis when undergoing surgery for malignant abdominal or pelvic tumor. Therefore, this study was conducted to determine the proportion of patients receiving thromboprophylaxis among those undergoing surgery for malignant abdominal or pelvic tumor. The secondary objectives were to determine the proportion of patients who develop symptomatic DVT, to evaluate the profile of the patients (in terms of age, gender, type of malignant tumor, stage of malignancy, and type of admitting hospital), and to determine the reasons for not giving thromboprophylaxis.

## Methods

This prospective multicenter observational study (trial registration no: CTRI/2013/05/003617) was conducted in accordance with the principles laid by the 18th World Medical Assembly (Helsinki, 1964) and all subsequent amendments and guidelines for Good Epidemiology Practice. Each participating site ensured that all necessary regulatory submissions (e.g., independent ethics committee) were performed in accordance with local regulations including local data protection regulations.

### Eligibility Criteria

Adults (≥ 18 years of age), who were hospitalized for abdominal or pelvic malignant tumor (such as colon, rectum, stomach, liver, pancreas, prostate, urinary bladder, uterus, and ovary) and had undergone surgery for this malignant tumor in the preceding 2 days, were included after undertaking an informed consent. Patients, who had a life expectancy of less than 1 month and were participating in another clinical trial, were excluded.

### Data Collection and Validation

The recruitment period was 11 months. The data were recorded prospectively for individual patients during the follow-up period of either 30 ± 7 days or at discharge, whichever was later.

During the course of the study, the investigator assessed the patient for clinical signs of DVT such as pain, swelling, and redness of the leg and dilatation of the surface veins. If the patient showed signs, he/she was evaluated for symptomatic DVT using Doppler ultrasonography.

At baseline visit, details of abdominal or pelvic cancer such as type of cancer, metastasis [14], and anticancer treatment employed; and details of DVT prophylaxis like duration and type of thromboprophylaxis, and reasons for not giving DVT prophylaxis, were recorded. At the mandatory last visit (i.e. at discharge or at 30 ± 7 days, whichever was later), details of DVT prophylaxis and reasons for not giving DVT prophylaxis were recorded. Data were collected using electronic case report forms.

### Statistical Analysis

Determination of sample size: Assuming that 44% of the patients would be given thromboprophylaxis [13], with 90% confidence interval (CI) and 5% precision, 265 subjects were required for this study. Considering a drop-out of 10%, it was planned to recruit 295 subjects.

Statistical methods: Cochran–Mantel–Haenszel test and chi-squared test were used to make comparisons between groups. A *p* value < 0.05 was considered as significant.

## Results

Among the 13 recruited investigators, 11 were surgical oncologist, 1 was a medical oncologist, and 1 was a surgical gastroenterologist. They were recruited from different geographical locations in India—1 from the north, 2 from west, 3 from east, and the remaining 7 from the south.

### Patient Disposition

Between December 2012 and November 2013, a total of 306 patients were enrolled. Out of 306 patients, 300 patients were considered for analysis as 6 patients did not meet the eligibility criteria (5 patients did not sign the informed consent form within 2 days, and the histopathology report showed a benign ovarian mass for 1 patient). Out of 300 patients, 298 patients (99.3%) completed the study, and 2 patients (0.7%) died during the course of the study.

### Baseline Characteristics

Of 300 patients, 217 (72.3%) were women. The mean ± standard deviation (SD) age was 53.2 ± 12.2 years. Ovarian cancer (*n* = 79, 26.3%) was the most common type of cancer. Among the stages of cancer, stage I (*n* = 70, 23.3%) was the most common. The most frequently (*n* = 23, 7.7%) observed metastasis was of “other” type which included metastasis resulting in rectovaginal fistula or ascites, periaortic (around abdominal aorta), and periportal (around portal vein) metastasis, supraclavicular, para-aortic lymph nodes, and metastasis to peritoneum, cervix, omentum, lymph node, rectum, endometrium, and caecum. In total, 63 (21.0%) patients had received chemotherapy, 18 (6.0%) patients had received radiation, 10 (3.3%) patients had undergone surgery (prior to this hospitalization), and 2 (0.7%) patients had taken hormonal therapy. Some patients had taken more than one therapy prior to this study. The mean ± SD duration of cancer was 1.2 ± 0.7 years. (Table 1).

**Table 1:** Baseline characteristics

	Total, *N* = 300
Age	
Mean (SD)	53.2 (12.2)
Median (Min–max)	54.0 (18–81)
Female	217 (72.3)
Type of cancer	
Ovarian cancer	79 (26.3)
Gastric cancer	46 (15.3)
Cervix carcinoma	44 (14.7)
Colon cancer	35 (11.7)
Rectal cancer	33 (11.0)
Uterine cancer	25 (8.3)
Bladder cancer	14 (4.7)
Endometrial cancer	6 (2.0)
Pancreatic carcinoma	5 (1.7)
Prostate cancer	4 (1.3)
Esophageal carcinoma	2 (0.7)
Renal cell carcinoma	2 (0.7)
Small intestine carcinoma	2 (0.7)
Gall bladder cancer	1 (0.3)
Renal cancer	1 (0.3)
Vaginal cancer	1 (0.3)
Stage of cancer	
Stage 0	3 (1.0)
Stage I	70 (23.3)
Stage II A	62 (20.7)
Stage II B	39 (13.0)
Stage II C	13 (4.3)
Stage III	31 (10.3)
Stage III A	16 (5.3)
Stage III B	18 (6.0)
Stage III C	25 (8.3)
Stage IV	19 (6.3)
Stage IV A	2 (0.7)
Stage IV B	2 (0.7)
Metastasis present	31 (10.3)
^a^Other	23 (7.7)
Liver	5 (1.7)
Pelvis	2 (0.7)
Pleura	1 (0.3)
Lung	0
Brain	0
Bone	0
Mediastinum	0
^b^Previous anticancer therapy	
Chemotherapy	63 (21.0)
Radiation	18 (6.0)
Surgery (prior to this hospitalization)	10 (3.3)
Hormonal therapy	2 (0.7)
Duration of cancer (years)	
Mean (SD)	1.2 (0.7)
Median (Min–max)	1.1 (1.0–10.2)

Abbreviations: *SD*, standard deviation; *Min–max*, minimum–maximum

^a^The other types of metastasis included malignant mixed mullerian tumor of uterus and pelvic peritoneum, rectovaginal fistula due to metastasis, ascites due to metastasis, periaortic (around abdominal aorta) and periportal (around portal vein) metastasis, supraclavicular, para-aortic lymph nodes and metastasis to peritoneum, cervix, omentum, lymph node, and rectum

^b^One patient may have more than one previous anticancer therapy

Percentages have been calculated using *N* as the denominator

All values represent *n* (%) unless specified

Duration of cancer (years) = (baseline visit date − date of initial diagnosis of cancer) + 1

### Proportion of Patients Who Were Given Thromboprophylaxis

Of the 300 patients, 162 (54%, 90% CI: 0.49–0.59) patients received thromboprophylaxis either at baseline visit or at 30-day visit or at discharge, while 138 patients did not receive thromboprophylaxis. Out of 162 patients who received thromboprophylaxis, most (*n* = 100; 61.7%) patients received it for ≤7 days, while approximately one-fourth of the patients (42 [25.9%]) received thromboprophylaxis for 8–15 days (online resource: Supplementary Table 1). Seventy-one (43.8%) patients received thromboprophylaxis only at baseline; 4 (2.5%) patients received it only at discharge; 77 (47.5%) patients received at baseline and discharge; and 10 (6.2%) patients received at baseline, discharge, and at 30-day visit. Overall, 78/162 (48.1%) patients were given only pharmacological thromboprophylaxis, 27/162 (16.7%) patients received only mechanical thromboprophylaxis, and 57/162 (35.2%) patients received both pharmacological and mechanical thromboprophylaxis. LMWH (128/162, 79.0%) and graduated compression stockings (74/162, 45.7%) were the most commonly used pharmacological and mechanical thromboprophylaxis, respectively. (Table 2).

**Table 2:** Type of thromboprophylaxis

	Total number of patients on thromboprophylaxis, *N* = 162
Pharmacological thromboprophylaxis *n*_1_ = 135	
Low-molecular-weight heparin	128 (79.0)
*Enoxaparin sodium	65 (48.1)
*Dalteparin sodium	61 (45.2)
*Nadroparin calcium	6 (4.4)
*Parnaparin	3 (2.2)
Unfractionated heparin	8 (4.9)
Mechanical thromboprophylaxis, *n*_2_ = 84	
Graduated compression stocking	74 (45.7)
Active and passive limb exercise	3 (1.9)
Intermittent pneumatic compression	1 (0.6)
Active and passive limb exercise, early ambulation	1 (0.6)
Active mobilization	1 (0.6)
Ambulation	1 (0.6)
Ambulatory measures	1 (0.6)
Leg exercise—passive manual compression	1 (0.6)
Leg physio	1 (0.6)
Limb exercise	1 (0.6)
Limb exercise and early mobilization	1 (0.6)
Venous foot pump	0

Percentages marked with “*” are calculated with reference to *n*_1_

Percentages other than those marked with “*” are calculated with reference to *N*

All values represent *n* (%)

For dalteparin and dalteparin sodium, they are the same medication (active ingredient) and combined into dalteparin sodium

For enoxaparin and enoxaparin sodium, they are the same medication (active ingredient) and combined into enoxaparin sodium

There were 57 patients who were on both pharmacological and mechanical thromboprophylaxis

A subject can be given more than one category of pharmacological/mechanical thromboprophylaxis

The above values are based on unique subject counts

### Proportion of Patients Who Developed Symptomatic Deep Vein Thrombosis

None of the patients assessed at the end of 30 days or at discharge (after 30 days) had symptoms of DVT.

### Evaluation of the Profile of Patients Who Were Given Thromboprophylaxis

Among the age groups analyzed, the age group of ≥ 60 years had the highest proportion (64/107; 59.8%) of patients receiving thromboprophylaxis. Overall, 50/83 (60.2%) men and 112/217 (51.6%) women enrolled received thromboprophylaxis. The most frequently reported type of cancer was ovarian cancer, and 40/79 (50.6%) patients suffering from ovarian cancer received thromboprophylaxis. Of the 299 patients who had cancer for 1–5 years, 161 (53.8%) patients received thromboprophylaxis; one patient had cancer for > 10 years and received thromboprophylaxis. Among patients with stage I and stage II A (the most frequently reported stages of cancer), 31/70 (44.3%) and 39/62 (62.9%), respectively, received thromboprophylaxis. Thromboprophylaxis was given in greater proportion of patients with metastasis than those without metastasis (61.3% vs 53.2%) (online resource: Supplementary Table 2).

### Reasons for Not Administering Thromboprophylaxis

For the 138 patients who did not receive thromboprophylaxis throughout the study, “patient was stable,” “the patient was stable and had no signs of DVT,” “early ambulation was encouraged,” and “risk of bleeding” were the major reasons for not administering pharmacological or mechanical thromboprophylaxis. (Table 3).

**Table 3:** Reasons for not administering thromboprophylaxis

	Patients not administered thromboprophylaxis, *N* = 138		
	Baseline	At discharge	At 1 month follow-up
Reasons for not administering pharmacological thromboprophylaxis			
Patient is stable	64 (46.4)	63 (45.7)	55 (39.9)
The patient was stable and had no signs of DVT	32 (23.2)	34 (24.6)	42 (30.4)
Early ambulation was encouraged	21 (15.2)	22 (15.9)	22 (15.9)
Risk of bleeding	19 (13.8)	16 (11.6)	15 (10.9)
Coagulopathy	1 (0.7)	1 (0.7)	–
Emergency surgery	1 (0.7)	–	–
Missing data	2 (1.4)	2 (1.4)	3 (2.2)
Reasons for not administering mechanical thromboprophylaxis			
Patient is stable	64 (46.4)	64 (46.4)	56 (40.6)
The patient was stable and had no signs of DVT	34 (24.6)	35 (25.4)	43 (31.2)
Early ambulation was encouraged	22 (15.9)	22 (15.9)	22 (15.9)
Not required (investigators discretion)	13 (9.4)	13 (9.4)	13 (9.4)
Emergency surgery	1 (0.7)	–	–
Missing data	4 (2.9)	3 (2.2)	3 (2.2)

Abbreviations: *DVT*, deep vein thrombosis

Percentages are calculated in reference to *N*

As patient 03–16 discontinued post-baseline, percentages do not add up to 100 for discharge and 1-month follow-up visits

Few patients have more than one reason for not administered thromboprophylaxis

All values represent *n* (%)

## Discussion

This prospective multicenter observational study showed that of the total 300 patients, who underwent surgery for malignant abdominal or pelvic tumor, approximately half of the patients received thromboprophylaxis. Of the patients who received thromboprophylaxis, nearly 48.1% received only pharmacological thromboprophylaxis, 16.7% received only mechanical, and 35.2% received both pharmacological and mechanical thromboprophylaxis. The proportion of patients, in whom thromboprophylaxis was given, was greater in men, in patients > 60 years, and in those with metastasis. LMWH and graduated compression stockings were the most commonly used thromboprophylaxis. Most patients who did not receive thromboprophylaxis were considered “stable” as per the investigator. None of the patients had symptoms of DVT.

The recommendations for thromboprophylaxis may vary slightly between different guidelines, but all major international guidelines like NCCN (National Comprehensive Cancer Network), ASCO, ESMO, and IMWG (International Multidisciplinary Working Group) broadly recommend postoperative thromboprophylaxis with LMWH for 7–10 days in cancer patients undergoing major surgery and for up to 4 weeks in cancer patients undergoing major abdominal or pelvic surgery [9–11]. In this study 54% patients received thromboprophylaxis which is higher than the 44% thromboprophylaxis rate seen in a multicenter prospective cohort study that included patients admitted in medical and surgical ICUs (Intensive care units) in India with at least two risk factors (not including sepsis) for DVT + ICU stay > 24 h or one risk factor (not sepsis) for DVT + ICU stay > 48 h [15]. However, the rate of thromboprophylaxis observed in this study was lower (54% vs 75.2%) than that observed in a retrospective medical record review in 10 teaching/community-based hospitals located in the United States [16]. Also the rate of pharmacological thromboprophylaxis in this study was lower than another prospective, observational study conducted between November 2009 and November 2010 in France in patients undergoing surgery for abdominal or pelvic cancer [12].

Of the patients who received thromboprophylaxis, LMWH was given in 79% of patients in this study. LMWHs are the preferred options over unfractionated heparin (UFH) for primary and secondary VTE prophylaxis on account of a number of benefits such as higher bioavailability (90% vs 30%), longer half-life (4–6 h vs 0.5–1 h), predictable and reproducible anticoagulant response, minimal interaction with non-anticoagulant related plasma proteins, and lesser propensity to cause heparin-induced thrombocytopenia and osteoporosis.

In our study, none of the patients had symptoms of DVT. Likewise, in another study conducted at Tata Memorial Hospital, India, between March 2002 and January 2004, in 99 patients undergoing surgery for colorectal cancer, no DVT was detected, and the study was terminated as the authors felt that the anticipated incidence of DVT may have been overestimated during sample size calculation [17]. In a retrospective study conducted at the Asian Medical Center, Seoul, South Korea, including 3645 patients who underwent colorectal cancer surgery between January 2006 and December 2008, 0.85% patients developed DVT [5]. However a multicenter, double-blind trial conducted at 10 university hospitals in Canada in patients undergoing colorectal cancer surgery showed a considerably higher DVT rate of 13.9% [18]. Genetic factors—like a low prevalence of the thrombophilic trait (known as factor V Leiden mutation) and thrombin gene *20210A*, low mean levels of fibrinogen, factor VIIc, and factor VIIIc—may contribute to the low prevalence of VTE in Asian population [19]. However, recent studies show that the incidences of symptomatic DVT and PE are comparable among Asian and Western population [5, 6]. Therefore, the reason for the absence of DVT in our study is not clear as it could either be attributed to a low prevalence of DVT in the study population or indicate adequacy of methods used for thromboprophylaxis and risk stratification or underscore a study limitation in terms of assessing the presence of only symptomatic DVT.

Among the three age groups in the study, the proportion of patients in whom thromboprophylaxis was given was highest for the age group of ≥ 60 years; this can be explained by the high risk of thrombosis observed in patients ≥ 60 years (2.63, 95% CI: 1.21–5.71) [20]. A total of 161/299 (53.8%) patients who had cancer for 1–5 years received thromboprophylaxis. Studies have shown that the risk of VTE is highest in the immediate period after diagnosis of cancer, with the adjusted odds ratio (OR) for developing VTE declining from 53.5 (95% CI: 8.6–334.3) in the first 3 months to 14.3 (95% CI: 5.8–35.2) in the period between 3 months and 1 year and to 3.6 (95% CI: 2.0–6.5) in between 1- and 3-year interval [2, 21]. Most patients who did not receive thromboprophylaxis in this study were considered “stable” as per the investigator or were those in whom early ambulation was encouraged or had contraindications like risk of bleeding.

### Strengths and Limitations of the Study

In view of the absence of studies determining the extent of thromboprophylaxis in Indian patients undergoing surgery for malignant abdominal or pelvic tumor, this study presents vital data on the thromboprophylaxis rate in these patients and also explores other key factors like the patient profile and primary reasons for not administering thromboprophylaxis. The study included patients from all four zones (north, south, east, and west) of India, and the 13 participating investigators varied on the basis of professional experience, thus providing diversity to the results. However, the patients were not evaluated for the presence of pulmonary embolism, which could be an important cause of death after discharge. Further, only patients having clinical signs of DVT were further evaluated using Doppler ultrasonography, and therefore, the study only assessed the presence of symptomatic DVT. Further studies to assess the presence of asymptomatic DVT in this population are required as 80% of DVT cases are asymptomatic [22].

## Conclusion

Cancer patients undergoing surgery represent very high risk for developing VTE and thromboprophylaxis is crucial in preventing VTE-related events. Physician education to increase adherence to international guidelines and strategies to promote compliance will go a long way in reducing morbidity and mortality in these patients.

## Supplementary Information

ESM 1 Supplementary Table 1 Duration of thromboprophylaxis, Supplementary Table 2. Patient profile (DOCX 18 kb).

## Declarations

### Data Availability

Qualified researchers may request access to patient-level data and related study documents including the clinical study report, study protocol with any amendments, blank case report form, statistical analysis plan, and dataset specifications. Patient-level data will be anonymized and study documents will be redacted to protect the privacy of trial participants. Further details on Sanofi's data sharing criteria, eligible studies, and process for requesting access can be found at https://www.clinicalstudydatarequest.com.
