# Supplementary material for: A Prospective Observational Study to Determine Rate of Thromboprophylaxis in Oncology Patients Undergoing Abdominal or Pelvic Surgery
Source: Indian J Surg Oncol. 2021 Mar 1;12(2):279–85. doi: 10.1007/s13193-021-01281-0 (PMC8272769; doi:10.1007/s13193-021-01281-0)
Supplement: Supplementary file 1 — Supplementary Table 1 Duration of thromboprophylaxis, Supplementary Table 2. Patient profile (DOCX 18 kb). [file 13193_2021_1281_MOESM1_ESM.docx]

**Supplementary Table 1. Duration of thromboprophylaxis­­**

| Duration on thromboprophylaxis  (days) | Patients on only pharmacological  thromboprophylaxis  N = 78 | Patients on only mechanical  thromboprophylaxis  N = 27 | Patients on both  pharmacological and  mechanical  thromboprophylaxis  N = 57 | Patients on  any thromboprophylaxis  N = 162 |
| --- | --- | --- | --- | --- |
| ﻿≤ 7 | 67 (85.9) | 19 (70.4) | 14 (24.6) | 100 (61.7) |
| 8 –15 | 10 (12.8) | 6 (22.2) | 26 (45.6) | 42 (25.9) |
| 16 – 30 | 1 (1.3) | 0 | 7 (12.3) | 8 (4.9) |
| >30 | 0 | 2 (7.4) | 10 (17.5) | 12 (7.4) |

Percentages are calculated in reference to N.

All values represent n (%)

**Supplementary Table 2. Patient profile**

|  | N=300 | On thromboprophylaxis n (%) | Not on thromboprophylaxis  n (%) |
| --- | --- | --- | --- |
| Age (years) |  |  |  |
| <40 | 46 | 25 (54.3) | 21 (45.7) |
| ≤40 to <60 | 147 | 73 (49.7) | 74 (50.3) |
| ≥60 | 107 | 64 (59.8) | 43 (40.2) |
| Gender |  |  |  |
| Female | 217 | 112 (51.6) | 105 (48.4) |
| Type of cancer |  |  |  |
| Ovarian cancer | 79 | 40 (50.6) | 39 (49.4) |
| Gastric cancer | 46 | 30 (65.2) | 16 (34.8) |
| Cervix carcinoma | 44 | 14 (31.8) | 30 (68.2) |
| Colon cancer | 35 | 20 (57.1) | 15 (42.9) |
| Rectal cancer | 33 | 18 (54.5) | 15 (45.5) |
| Uterine cancer | 25 | 17 (68.0) | 8 (32.0) |
| Bladder cancer | 14 | 10 (71.4) | 4 (28.6) |
| Endometrial  cancer | 6 | 2 (33.3) | 4 (66.7) |
| Pancreatic  carcinoma | 5 | 2 (40.0) | 3 (60.0) |
| Prostate cancer | 4 | 4 (100) | 0 |
| Oesophageal  carcinoma | 2 | 1 (50.0) | 1 (50.0) |
| Renal cell  carcinoma | 2 | 1 (50.0) | 1 (50.0) |
| Small intestine  carcinoma | 2 | 0 | 2 (100) |
| Gallbladder cancer | 1 | 1 (100) | 0 |
| Renal cancer | 1 | 1 (100) | 0 |
| Vaginal cancer | 1 | 1 (100) | 0 |
| Stage of cancer |  |  |  |
| ﻿ Stage 0 | 3 | 0 | 3 (100) |
| Stage I | 70 | 31 (44.3) | 39 (55.7) |
| ﻿ Stage II A | 62 | 39 (62.9) | 23 (37.1) |
| ﻿ Stage II B | 39 | 20 (51.3) | 19 (48.7) |
| Stage II C | 13 | 8 (61.5) | 5 (38.5) |
| Stage III | 31 | 18 (58.1) | 13 (41.9) |
| Stage III A | 16 | 9 (56.3) | 7 (43.8) |
| Stage III B | 18 | 11 (61.1) | 7 (38.9) |
| Stage III C | 25 | 14 (56.0) | 11 (44.0) |
| Stage IV | 19 | 10 (52.6) | 9 (47.4) |
| Stage IV A | 2 | 1 (50.0) | 1 (50.0) |
| Stage IV B | 2 | 1 (50.0) | 1 (50.0) |
| Duration of cancer (years) |  |  |  |
| ﻿ <1 | 0 | 0 | 0 |
| 1 – 5 | 299 | 161 (53.8) | 138 (46.2) |
| 5 – 10 | 0 | 0 | 0 |
| >10 | 1 | 1 (100) | 0 |
| Presence of metastasis |  |  |  |
| Yes | 31 | 19 (61.3) | 12 (38.7) |
| No | 269 | 143 (53.2) | 126 (46.8) |
| Location of hospital |  |  |  |
| Urban | 263 | 136 (51.7) | 127 (48.3) |
| Rural | 37 | 26 (70.3) | 11 (29.7) |
| Type of hospital |  |  |  |
| Corporate | 136 | 74 (54.4) | 62 (45.6) |
| Any other | 75 | 32 (42.7) | 43 (57.3) |
| Nursing Home | 69 | 45 (65.2) | 24 (34.8) |
| Government | 20 | 11 (55.0) | 9 (45.0) |
| Attachment to academic institution |  |  |  |
| Yes | 37 | 28 (75.7) | 9 (24.3) |
| No | 263 | 134 (51.0) | 129 (49.0) |
